# Improving the Thermostability of Acidic Pullulanase from *Bacillus naganoensis* by Rational Design

**DOI:** 10.1371/journal.pone.0165006

**Published:** 2016-10-20

**Authors:** Meihui Chang, Xiaoyu Chu, Jinzhi Lv, Qingbin Li, Jian Tian, Ningfeng Wu

**Affiliations:** 1 Biotechnology Research Institute, Chinese Academy of Agricultural Sciences, Beijing, 100081, China; 2 Institute of Food Science and Engineering, Jiangxi Agricultural University, Nanchang, 330045, China; 3 College of Biological Sciences, China Agricultural University, Beijing, 100094, China; Russian Academy of Medical Sciences, RUSSIAN FEDERATION

## Abstract

Pullulanase (EC 3.2.1.41) plays an important role in the specific hydrolysis of branch points in amylopectin. Enhancing its thermostability is required for its industrial application. In this study, rational protein design was used to improve the thermostability of PulB from *Bacillus naganoensis* (AB231790.1), which has strong enzymatic properties. Three positive single-site mutants (PulB-D328H, PulB-N387D, and PulB-A414P) were selected from six mutants. After incubation at 65°C for 5 min, the residual activities of PulB-D328H, PulB-N387D, and PulB-A414P were 4.5-, 1.7-, and 1.47-fold higher than PulB-WT, and their *T*_m_ values (the temperature at which half protein molecule denature) were 1.8°C, 0.4°C, and 0.9°C higher than PulB-WT, respectively. Then the final combined mutant PulB-328/387/414 was constructed. The t_1/2_ of it was 12.9-fold longer than that of PulB-WT at 65°C and the total increase in *T*_m_ of it (5.0°C) was almost 60% greater than the sum of individual increases (3.1°C). In addition, kinetic studies revealed that the *k*_cat_ and the *k*_cat_/*K*_m_ of PulB-328/387/414 increased by 38.8% and 12.9%. The remarkable improvement in thermostability and the high catalytic efficiency of PulB-328/387/414 make it suitable for industrial applications.

## Introduction

Natural starch is one of the most familiar carbohydrates and is abundant in foods such as potatoes, corn, cassava, and wheat. Starch is mainly composed of α-1,4-glycosidic bonds and α-1,6-glycosidic bonds, with α-1,6-glycosidic bonds forming amylopectin [1]. Generally, starch contains 20–25% amylose and 75–80% amylopectin. The starch in waxy maize and glutinous rice is composed almost entirely of amylopectin. Because most amylolytic enzymes are specific for α-1,4-glucosidic bonds, the α-1,6-glucosidic bonds must be effectively cleaved for complete utilization of amylopectin.

Pullulanase (EC 3.2.1.41) is a well-known starch-debranching enzyme that has the specific ability to hydrolyze α-1,6–glycosidic bonds in amylopectin to obtain amylose [1,2]. It belongs to glycosyl hydrolase (GHase) family 13 (also known as the α-amylase family). Pullulanase has great commercial promise in starch saccharification to produce high-glucose syrup from starch [2,3], and can also be used in the production of fuel ethanol, low-calorie beer, resistant starch, and maltotriose [4–6]. Currently, the saccharification reaction is usually performed at 60–70°C and pH 4.5 to maximize the activity of glucoamylase [7,8]. Thus, a pullulanase with matching optimum pH, optimum temperature, and sufficient stability under high temperature and sub-acid conditions would be of significant value.

To date, many pullulanase genes from different species have been found, cloned, and expressed in heterologous expression systems, including those from *Anaerobranca gottschalkii* [9], *Bacillus naganoensis* [10,11], *B*. *deramificans* [12,13], *B*. *acidopullulyticus* [14,15], *Geobacillus thermoleovorans NP33*[16], *Anoxybacillus* species [17–19], *Thermotoga petrophila* [20], *Exiguobacterium sp*. *SH3* [21], and *Klebsiella variicola* [22]. As the protein rational design based on multi-sequence alignments is a very efficient method for the directed evolution of functional enzymes, some efforts toward improving the stability or catalytic efficiency of pullulanase have achieved success [23–25]. For example, through site-directed mutagenesis, the thermostability of Pul-D437H and Pul-D503Y from *B*. *deramificans* was improved [12], and by N-terminal truncation, the thermostability of D437H/D503Y-T_1_ was further improved [26]. Using four data-driven rational design methods (B-FITTER, proline theory, PoPMuSiC-2.1, and the sequence consensus approach), the thermostability of Pul-E518I-S662R-Q706P from *B*. *acidopullulyticus* was markedly improved [14]. Using structure and sequence analysis-based engineering, the thermostability of the mutant M18 (Y477A/D3/Y175C/L215P/R473E) Type I pullulanase, PulA, from *Anoxybacillus sp*. LM18–11 was significantly improved [19]. And based on conserved sites and homology modeling analysis, the half-lives of F581L and F581Q were increased by 4.20 and 3.70 min compared to the wild-type (WT) pullulanase of *Klebsiella variicola* [22].

Accoding to the literatures and research studies, PulB from *B*. *naganoensis* (GenBank accession number AB231790.1), with an optimum temperature of 60°C and optimum pH 4.5 [10,11], meets the criteria necessary for saccharification. And PulB from *B*. *naganoensis* has better catalytic activity [27–29]. But by preliminary trial, it shows good stability under acidic conditions but not under thermal stress, limiting its further application. Therefore, modifying the *pulB* gene by rational design to improve the thermal stability of PulB is a very urgent and important endeavor.

In this study, we collected four different Pul sequences, which were highly similar to PulB sequences from *B*. *naganoensis*, but with better thermostability than PulB. Through homology modeling, sequence alignment, and conserved site analysis, we performed site-specific mutation of *pulB* to achieve significant improvements in the thermostability of the enzyme.

## Materials and Methods

### Bacterial strains, plasmids, restriction enzymes, and chemicals

*B*. *naganoensis* (GenBank accession number AB231790.1) was purchased from Germany (DSMZ, German Collection of Microorganisms and Cell Cultures; Braunschweig, Germany). *Escherichia coli* mach1-T1 (Tiangen Biotech; Beijing, China) and the pGM-T vector (Tiangen) were used for genetic manipulation of recombinant plasmids. *E*. *coli* BL21 (DE3) (Novagen; Darmstadt, Germany) and the pET-22b(+) vector (Novagen), which introduces a His6-tag (His-tag™; Novagen) at the N-terminus, were used for protein expression. All of the restriction enzymes and T4 Ligase were from New England Biolabs (Ipswich, MA, USA). dNTPs, Easy Taq, Fast Pfu polymerase, and recombinase were purchased from TransGen Biotech (Beijing, China). Differential Scanning Calorimetry (DSC, GE Healthcare Life Science; Pittsburgh, PA, USA) was located in feed research institute (Chinese Academy of Agricultural Sciences [CAAS]; Beijing, China). All of the chemicals were analytical grade.

### Databases and bioinformatic tools

Protein coding sequences were retrieved from the NCBI GenBank Database. Homology modeling of the enzymes and sequences alignment analysis were performed using Discovery Studio-2.5 software [30].

### Construction of WT and mutants

The *PulB*-WT gene of pullulanase was cloned in the pET22b vector using the *B*. *naganoensis* (AB231790.1) genome as template DNA and pul-WT-F and pul-WT-R (S1 Table) as primers [31]. Six one-site mutants and two multipoint mutants were generated through homologous recombination using the primers listed in S1 Table [32]. All of the mutants generated from homologous recombination of fragment 1 (using primers pul-F and pET22b-R) and fragment 2 (using primers pul-R and pET22b-F), using the pET22b-*pul*-WT plasmid as template DNA. To verify incorporation of the mutant genes, DNA sequencing was performed at the State Key Laboratory of Crop Genetic Improvement (CAAS). The verified plasmids were transformed into competent *E*. *coli* BL21 (DE3) cells for expression.

### Heterologous expression and purification

The recombinant strains were grown in Luria–Bertani medium supplemented with 50 μg/mL ampicillin shaking at 200 rpm at 37°C to an OD_600_ of 0.6, after which IPTG was added to a final concentration of 0.4 mM. After incubation for 20 h at 16°C, the cells were collected by centrifugation at 8000 rpm for 10 min and resuspended in 20 mM Tris/HCl buffer (pH 7.0) to be broken by the ultrasonicator. The supernatant of ultrasonically broken cells was obtained after centrifugation at 12000 rpm for 20 min. Then the recombinant proteins labeled with a His_6_-tag were purified on a Ni–NTA His-bind resin column (Novagen). As the protein extracts obtained from this process exhibited a high concentration of imidazole, they were dialyzed using 20 mM Tris/HCl buffer (pH 7.0). The proteins were concentrated by polyethylene glycol 8000 (PEG 8000) [33], and the purified proteins were used to evaluate thermostability variation and kinetic parameters.

### Standard enzyme assay

The enzyme assay method was improved according to the protocol by Cheng [34]. Briefly, 250 μL diluted enzyme solution was added to 500 μL preheated buffer (pH 4.5 citric acid-phosphate) containing 0.5% pullulan sugar (WAKO, Japan), and then incubated for 20 min at 60°C. The reaction was terminated by the addition of 750 μL dinitrosalicylic acid solution (DNS), followed by incubation in a boiling water bath for 10 min and rapid cooling. The absorbance was measured at 550 nm. The negative control had 250 μL buffer instead of enzyme solution, with all of the other reaction conditions being the same. Standard glucose samples of various concentrations were measured using the DNS method. Based on the amount of hydrolysis product (reducing sugar), enzyme activity was calculated. One unit of pullulanase activity is defined as the amount of enzyme liberating 1 μmol of reducing sugar per min at 60°C and pH 4.5 [35].

### Thermostability assay

To determine the thermostability of enzymes, the enzymes were diluted to the same concentration, and then incubated at 60°C or 65°C for 0 min, 2 min, 5 min, 10 min, 20 min, 30 min, 40 min, 50 min, 60 min, respectively, and rapidly cooled. Finally, the residual activity was measured using the standard assay. The activity of the enzyme without incubation was defined as 100%. *T*_m_ values [the temperature at which half protein molecule denature] were determined by differential scanning calorimetry (DSC) as previously described [36, 37]. The samples were diluted to 0.2 mg mL^−1^ in 20 mM Tris/HCl buffer (pH 7.0) and heated from 40°C to 105°C at a rate of 2°C min^−1^. A nitrogen purge cell was used to maintain a pressure of 0.413 MPa (Nano-DSC, GE Healthcare).

### Kinetic parameters of WT and mutant enzymes

The kinetic parameters of WT and the mutant enzymes were determined at the optimal temperature of 60°C and optimal pH 4.5 using 0, 0.5, 1.0, 1.5, 2.0, 2.5, 3.0, 3.5, 4.0, 4.5, 5.0, 6.0, 8.0, 10.0, 12.5, 15.0 and 17.5 mg mL^−1^ pullulan sugar [38]. Each enzyme activity assay was performed with at least three replicates. The steady-state kinetic parameters *K*_m_ and *k*_cat_ of the PulB-WT and mutants were determined by fitting the Michaelis–Menten equation to the initial velocity data using GraphPad Prism (GraphPad Software Inc; La Jolla, CA, USA).

## Results

### Homology modeling of enzymes and DNA sequence analysis

**Fig 1:**
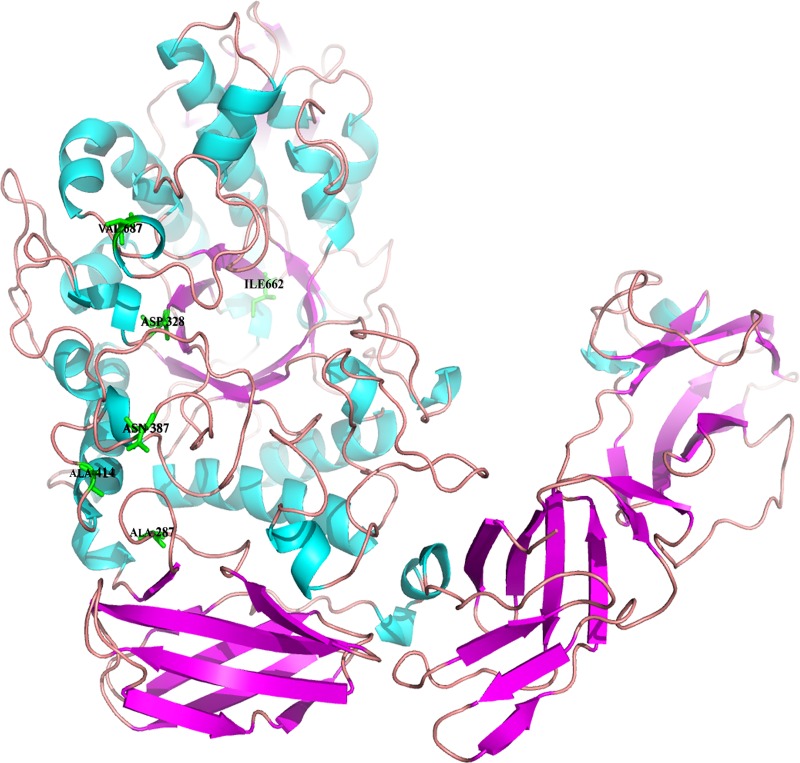
The 3D-model of PulB-WT.

We selected four Pul protein from different species (*B*. *acidopullulyticus* [39], *B*. sp. CICIM 263 [40], *T*. *maritima* MSB8 [41], and *T*. *neapolitana* [42]), which had better thermostability naturally. Through homology modeling, sequence alignment and conserved site analysis, we found that some portions of these four amino acid sequences were absolutely conserved. Their sequence identities with PulB from *Bacillus naganoensis* were 64.0%, 38.6%, 44.0% and 43.9%, respectively. There were 165 residues absolutely consistent among the five homologous enzymes. And there were 19 residues deviation only in the PulB from *B*. *naganoensis*. Using a rational design method of protein design, only six residues located at the core of PulB-WT were mutated, because the regions in the N- and C- terminal of the enzyme PulB-WT were not the important functional regions, as shown in Fig 1 and S1 Text.

### Protein purification and thermostability assays of WT and the six mutants

**Fig 2:**
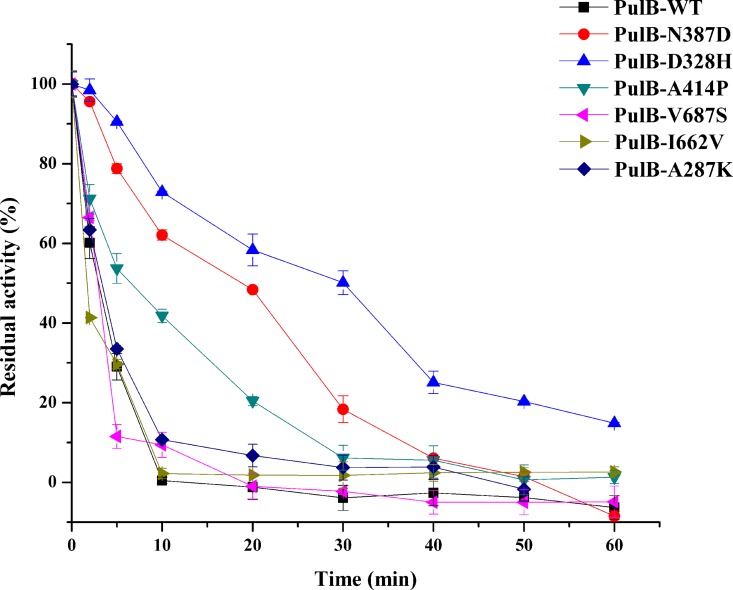
The thermostability assays of the PulB-WT and its mutants (PulB-A287K, PulB-D328H, PulB-N387D, PulB-A414P, PulB-I662V, and PulB-V687S), after incubation at 60°C for different minutes. Enzymatic activity was assayed using the standard enzyme assay. Data points correspond to the mean values of three independent experiments.

The genes encoding WT and mutant enzymes were cloned and expressed in *E*. *coli* BL21 (DE3). After purification by Ni-affinity chromatography, each of the expressed proteins migrated as a single band in SDS-PAGE, and there was an obvious band of each protein at about 100 kDa (S1 Fig). The optimum pH was 4.5 and optimal temperature was 60°C for PulB-WT (S2 Fig), consistent with referenced pullulanase from *B*. *naganoensis* [43]. It showed good pH stability, more than 80% of the activity was retained after incubation for 1 h under pH 4~9 at 37°C (S3 Fig). However, the PulB-WT showed poor themostability, only 30% of the activity was retained after incubation for 5min at 60°C (Fig 2). Among the mutants, we found that the thermostability of PulB-D328H was most significantly improved, followed by PulB-N387D and A414P third (Fig 2), whereas the thermostabilities of A287K, I662V, and V687S were worse than PulB-WT (Fig 2). We also tested the thermostability of PulB-D328H, N387D and A414P at 65°C, the result showed that their residual activities were respectively 4.5-, 1.7-, and 1.47-fold higher than PulB-WT incubated at 65°C for 5 min (S4 Fig). The *T*_m_ values of these three mutants were measured by DSC. Compared to PulB-WT (*T*_m_ 61.5±0.04°C), the *T*_m_ values of PulB-D328H, PulB-N387D, and PulB-A414P were 1.8°C, 0.4°C, and 0.9°C higher than PulB-WT, respectively (Table 1 and S5 Fig).

**Table 1:** *T*_m_ values of PulB-WT and one-site mutants.

	PulB-WT	PulB-D328H	PulB-N387D	PulB-A414P
***T***_***m***_ **value(°C)**	61.5±0.04	63.3±0.1	61.9±0.1	62.4±0.2

*T*_m_ values were determined by differential scanning calorimetry (DSC).

### Construction of two-site and three-site mutants and their thermostability assays

**Fig 3:**
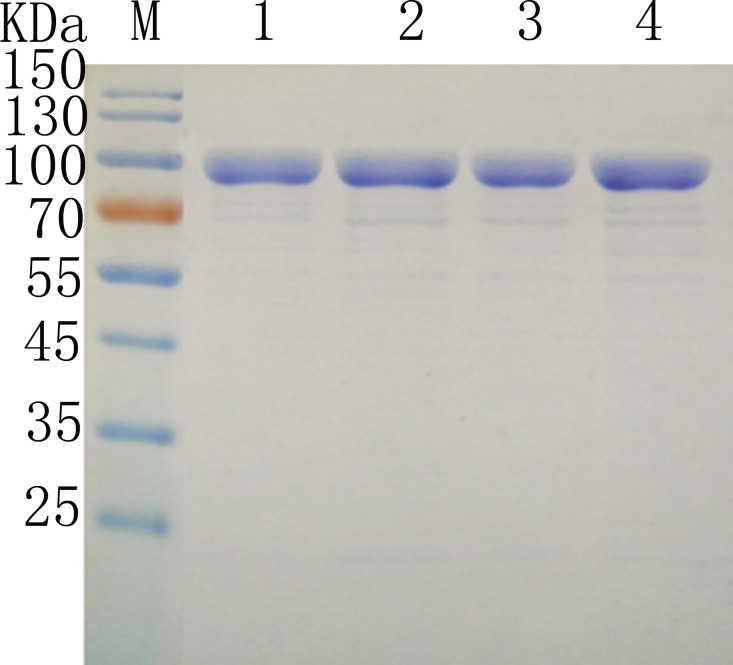
The SDS-PAGE of PulB-WT and different mutants. M, marker; lanes 1, 2, 3 and 4 was the purified protein of PulB-WT, PulB-D328H, PulB-328/387 and PulB-328/387/414, respectively.

**Fig 4:**
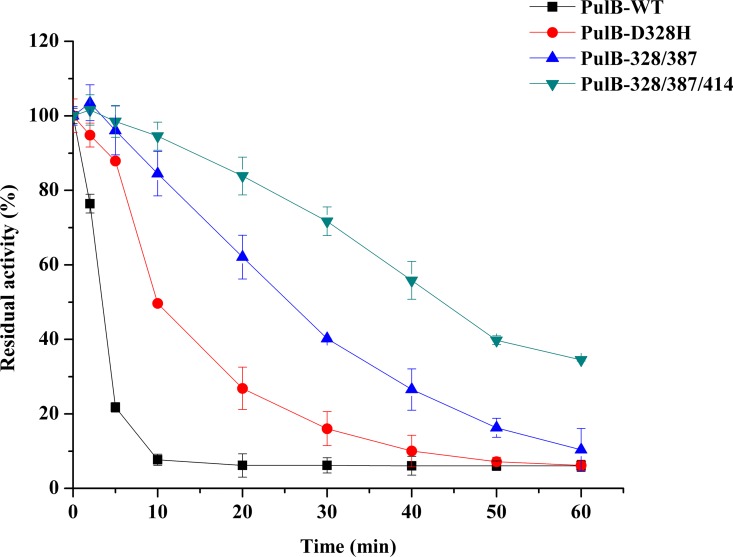
The thermostability assays of the PulB-WT and its mutants (PulB-D328H, PulB-328/N387, PulB-328/387/414), after incubation at 65°C for different minutes. Enzymatic activity was assayed using the standard enzyme assay. Data points correspond to the mean values of three independent experiments.

Single mutations for protein thermal stability often exist independently, but also exhibit a stacking effect [44]. To further improve the thermostability of pullulanase, a stepwise evolutionary approach based on iterative mutagenesis was designed [32, 45]. As the thermostability of D328H was the best, followed by N387D and A414P third, we constructed two-site mutant (PulB-328/387) based on D328H, and three-site mutant (PulB-328/387/414) based on PulB-328/387. The WT and mutants were expressed in *E*. *coli* BL21 (DE3) and purified by Ni-affinity chromatography, and the SDS-PAGE results of PulB-WT, PulB-D328H, PulB-328/387, and PulB-328/387/414 was shown in Fig 3. In thermostability assays, we found that PulB-328/387/414 had the best thermostability. After incubation at 65°C for 10 min, the residual activities of PulB-328/387, and PulB-328/387/414 were 11.1- and 12.4-fold higher than PulB-WT (Fig 4). The t_1/2_ for PulB-WT was about 3.5 min, while the t_1/2_ for PulB-328/387/414 was almost 45 min (Fig 3), being 12.9-fold longer than PulB-WT. These results were verified by DSC; compared to PulB-WT (*T*_m_ 61.5±0.04°C), the *T*_m_ of PulB-328/387/414 increased by 5.0°C and the *T*_m_ of PulB-328/387 was increased by 4.2°C (Table 2 and S6 Fig). The total increase in *T*_m_ of PulB-328/387/414 (5.0°C) was almost 60% greater than the sum of individual increases (1.8 + 0.4 + 0.9 = 3.1°C).

**Table 2:** *T*_m_ values of PulB-WT and combined mutants.

	PulB-WT	PulB-D328H	PulB-328/387	PulB-328/387/414
***T***_***m***_ **value(°C)**	61.5±0.04	63.3±0.1	65.7±0.1	66.5±0.2

*T*_m_ values were determined by differential scanning calorimetry (DSC).

### Kinetic parameters of WT and the mutants

The kinetic parameters of WT and the mutants were determined at the optimal temperature of 60°C and optimal pH 4.5 using 17 different substrate concentrations of pullulan sugar. The *k*_cat_ of one-site mutants (PulB-D328H, PulB-N387D, and PulB-A414P) were all higher than that of PulB-WT. Thus, the *k*_cat_/*K*_m_ of PulB-D328H was similar to that of PulB-WT; the *k*_cat_/*K*_m_ of PulB-N387D and PulB-A414P was higher than that of PulB-WT. With a similar *K*_m_ value, the *k*_cat_ of PulB-328/387 and PulB-328/387/414 were increased by 24.1% and 38.8%, respectively; therefore the *k*_cat_/*K*_m_ of PulB-328/387 and PulB-328/387/414 increased by 20.0% and 12.9%, respectively (Table 3). These results show that although these mutants exhibited improved thermostability, they did not necessarily affect the catalytic efficiency.

**Table 3:** The kinetic parameter of the PulB-WT and its mutants.

	*K*_m_(mg.ml^-1^)	*k*_cat_(min^-1^)	*k*_cat_/*K*_m_(ml.mg^-1^.min^-1^)
**PulB-WT**	1.22±0.11	43.36±0.87	35.54±3.92
**PulB-D328H**	1.79±0.15	64.64±1.24	36.17±3.65
**PulB-N387D**	1.84±0.16	73.72±1.62	40.07±4.36
**PulB-A414P**	1.68±0.17	73.57±1.72	43.79±5.45
**PulB-328/387**	1.27±0.11	53.81±0.97	42.54±4.53
**PulB-328/387/414**	1.50±0.09	60.20±0.91	40.13±3.01

## Discussion

Pullulanase is able to hydrolyze α-1,6–glycosidic bonds in amylopectin specifically. It would reduce the usage of glucoamylase, avoid the adverse reaction, and help to improve the conversion rate of starch in saccharification process. Because the industrial glucoamylase works at optimum temperature 60~65°C and pH 4.5±0.2, the property of pullulanase requires to be consistent with the working condition of glucoamylase. PulB from *B*. *naganoensis* with an optimum temperature of 60°C, optimum pH 4.5, and better catalytic activity, has terrific acid stability at pH 3~5 especially. However, its thermostability is poor (its t_1/2_ has only 3.5 min at 65°C). Therefore, in this study, we used a rational protein design method to improve the thermostability of the PulB.

The protein rational design based on multi-sequence alignments is a very efficient method for the directed evolution of functional enzymes. Many scientists successfully obtained desired enzymes by this method. To improve the thermal stability of PulB, we selected four Pul protein sequences which had better thermostability than PulB to do alignment analysis. Six residues were chosen to mutate among 19 residues which were only in the PulB of *B*. *naganoensis* differed from four thermostable Pul enzymes. Three positive mutants, PulB-D328H, PulB-N387D and PulB-A414P, were obtained. The reason of the thermostability improvement for PulB-D328H and PulB-N387D was probably because of the formation of strong ionic bond. At the 414 site on the loop section of PulB, when Ala was mutated to Pro, the rigidity of PulB-A414P was much greater than that of PulB-WT, aiding thermal stability, and many previous studies have verified that Pro can increase the rigidity and thermostability of enzymes [46, 47].

As the result, the t_1/2_ of the three-site mutant PulB-328/387/414 was 12.9-fold longer than that of PulB-WT at 65°C (Fig 4), and its *T*_m_ was increased by 5.0°C (Table 2). This is the first study to improve the thermal stability of PulB from *B*. *naganoensis* using homology modeling and conserved site analysis. Compared to Pul-D503Y/D437H from *B*. *deramificans*, the residual activity of Pul-D503Y/D437H was 83.1% after incubation at 65°C for 10 min [12], whereas the residual activity of PulB-328/387/414 remained 95.4% (Fig 4). The *T*_m_ of PulA-M18 (Y477A/D3/Y175C/L215P/R473E) from A. sp. LM18–11 increased by 4.00°C, and its residual activity was below 80% after incubation at 65°C for 20 min [19]. In comparison, PulB-328/387/414 exhibited 83.8% residual activity under the same conditions (Fig 4) and its *T*_m_ increased by 5.0°C (Table 2). The extent of the increase in PulB thermostability was outstanding and remarkable.

In conclusion, using rational design based on the sequence alignment guided consensus method, the best thermostable mutant PulB-328/387/414 was obtained. It exhibited a 5.0°C increase in *T*_m_, and its t_1/2_ was 12.9-fold longer than PulB-WT at 65°C. The *k*_cat_ and the *k*_cat_/*K*_m_ of it also increased by 38.8% and 12.9%. With remarkable thermostability and high catalytic efficiency, PulB-328/387/414 is well suited to industrial applications. This study provides an efficient method to improve the thermostability of enzymes that could be applied to other industrially relevant enzymes.

## Supporting Information

S1 Fig The SDS-PAGE of PulB-WT and different mutants. M, marker; lanes 1 and 2 was the supernatant of ultrasonically broken cells and the purified protein.
(TIF)

S2 Fig The enzymatic properties assay of PulB-WT. a: the optimal temperature assay of PulB-WT; b: the optimum pH assay of PulB-WT. Data points correspond to the mean values of three independent experiments.
(TIF)

S3 Fig The pH stability assays of the PulB-WT, after incubationat 37°C for 1 h under different pH (pH 2~10). Enzymatic activity was assayed using the standard enzyme assay. Data points correspond to the mean values of three independent experiments.
(TIF)

S4 Fig The thermostability assays of the PulB-WT and its mutants (PulB-D328H, PulB-N387D, PulB-A414P), after incubation at 65°C for different minutes. Enzymatic activity was assayed using the standard enzyme assay. Data points correspond to the mean values of three independent experiments.
(TIF)

S5 Fig *T*_m_ values assays of PulB-WT and one-site mutants by differential scanning calorimetry (DSC). (TIF)

S6 Fig *T*_m_ values assays of PulB-WT and combined mutants by differential scanning calorimetry (DSC). (TIF)

S1 Table Sequences of the primers used in this study. (DOCX)

S1 Text Sequence alignment between PulB-WT and *Bacillus acidopullulyticus*, *Bacillus sp*. CICIM 263, *Thermotoga maritima* MSB8, *Thermotoga neapolitana*. (PDF)
