# Supplementary figures and images for: Improving the Thermostability of Acidic Pullulanase from Bacillus naganoensis by Rational Design
Source: PLoS One. 2016 Oct 20;11(10):e0165006. doi: 10.1371/journal.pone.0165006 (PMC5072709; doi:10.1371/journal.pone.0165006)

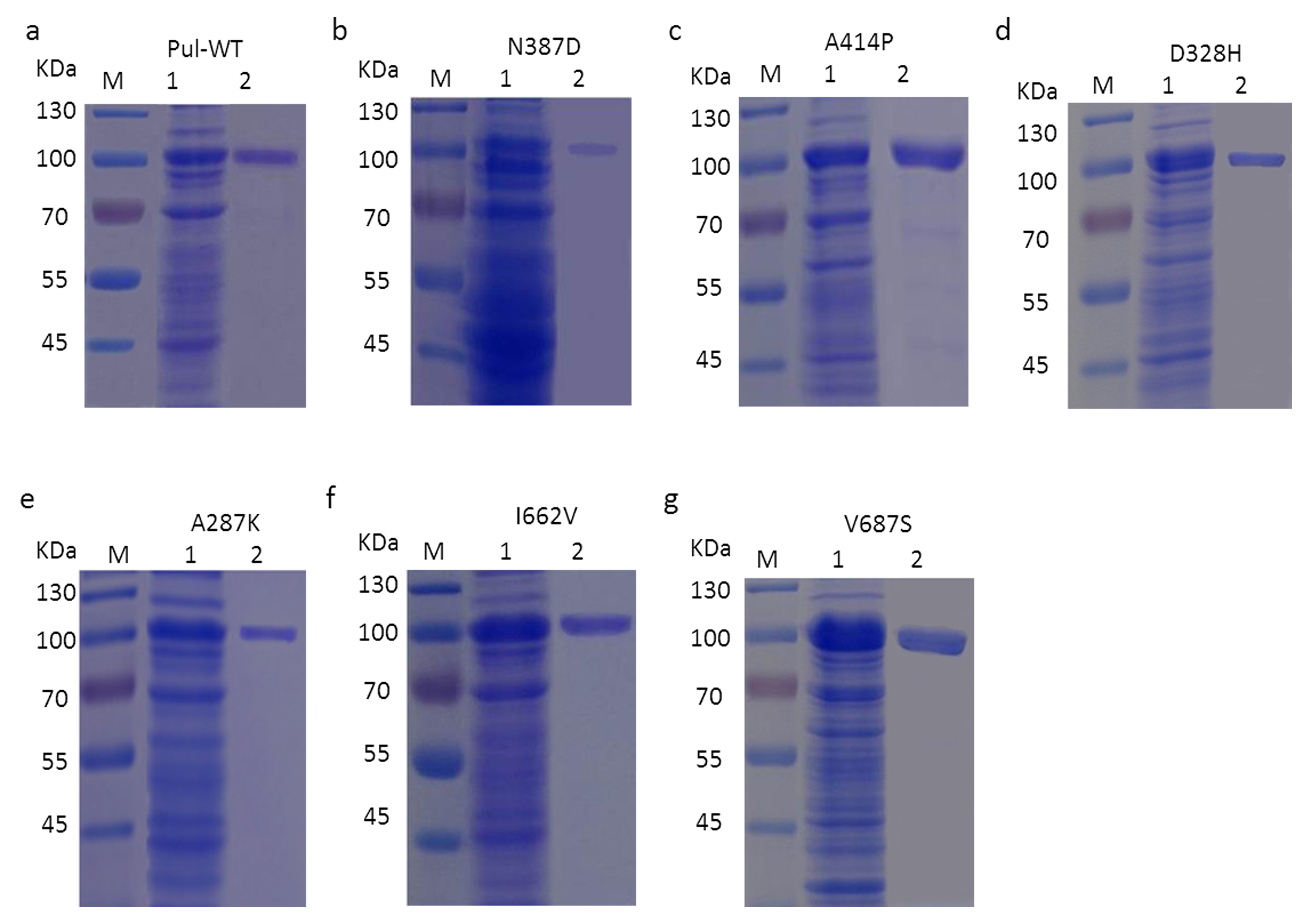

Supplement: S1 Fig — M, marker; lanes 1 and 2 was the supernatant of ultrasonically broken cells and the purified protein. (TIF) [file pone.0165006.s001.tif]

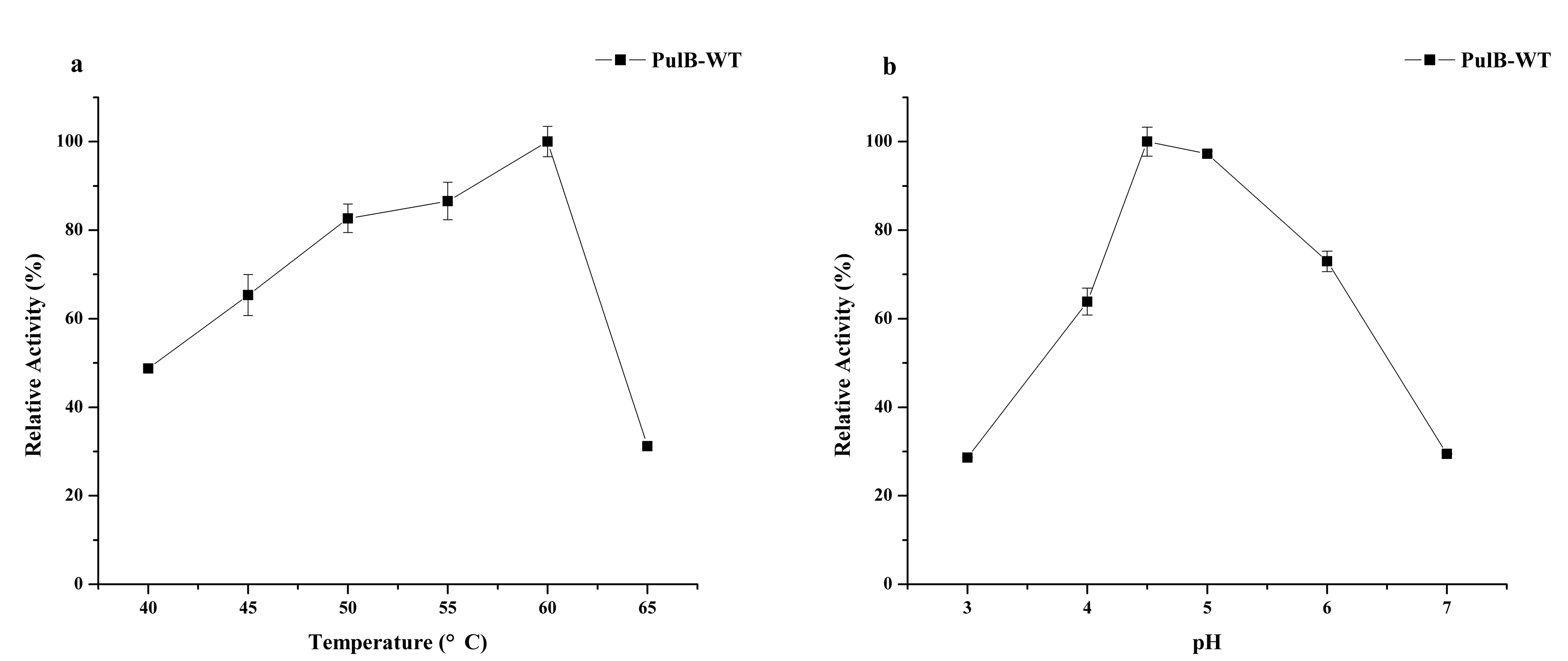

Supplement: S2 Fig — a: the optimal temperature assay of PulB-WT; b: the optimum pH assay of PulB-WT. Data points correspond to the mean values of three independent experiments. (TIF) [file pone.0165006.s002.tif]

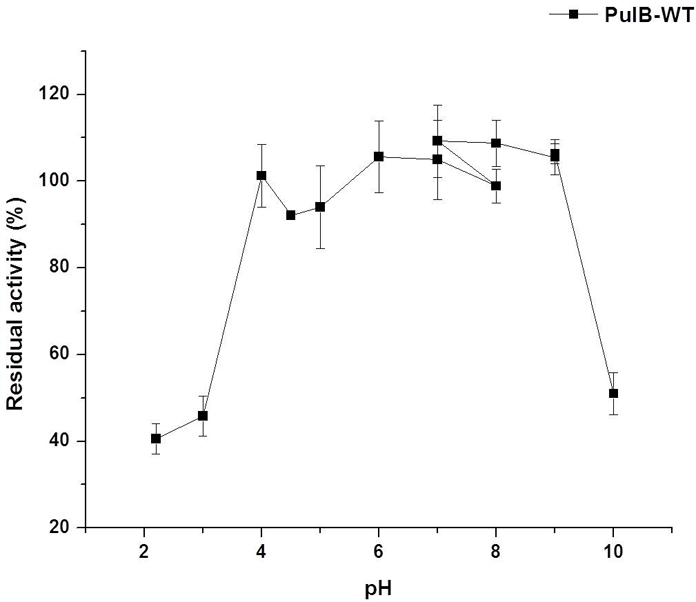

Supplement: S3 Fig — Enzymatic activity was assayed using the standard enzyme assay. Data points correspond to the mean values of three independent experiments. (TIF) [file pone.0165006.s003.tif]

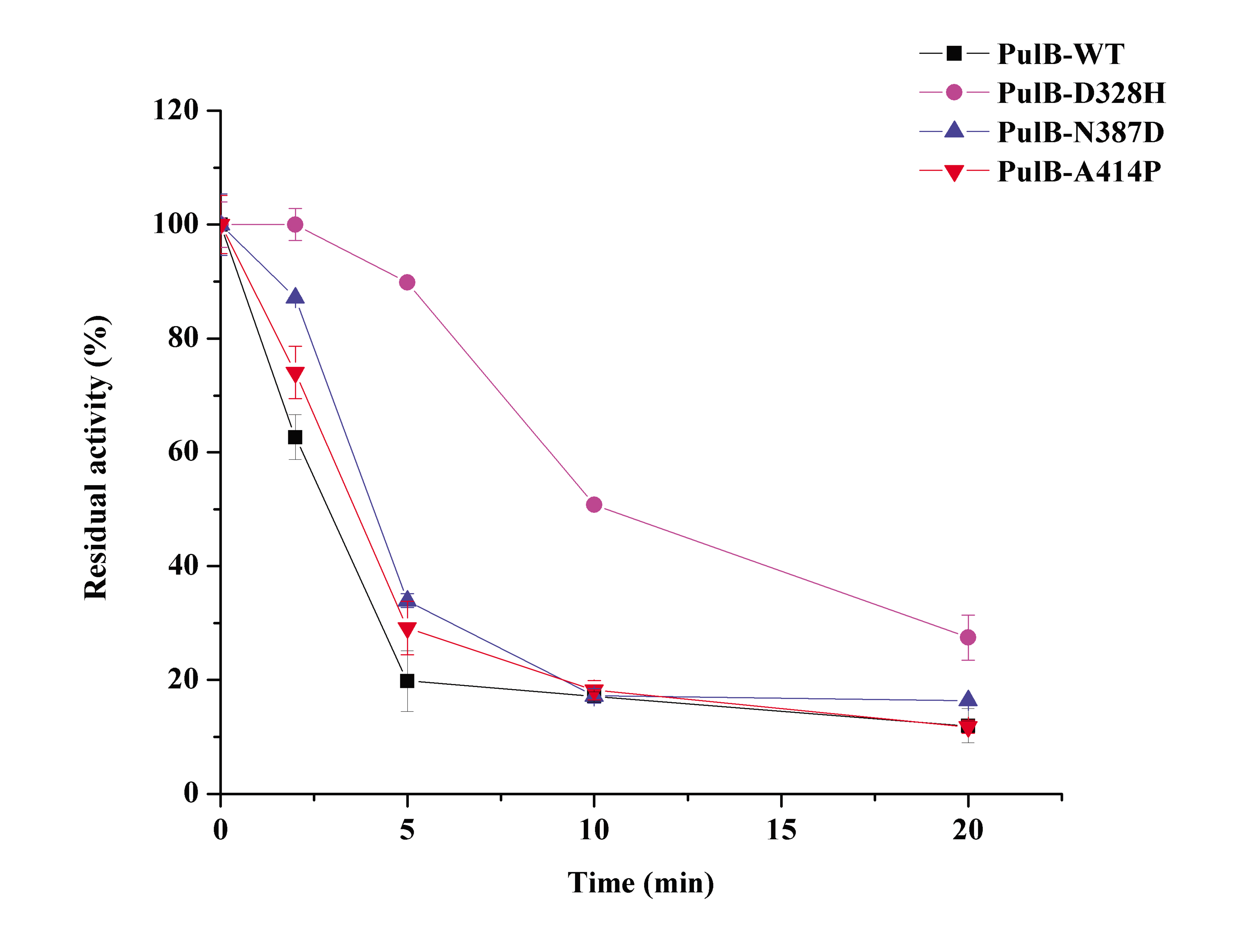

Supplement: S4 Fig — Enzymatic activity was assayed using the standard enzyme assay. Data points correspond to the mean values of three independent experiments. (TIF) [file pone.0165006.s004.tif]

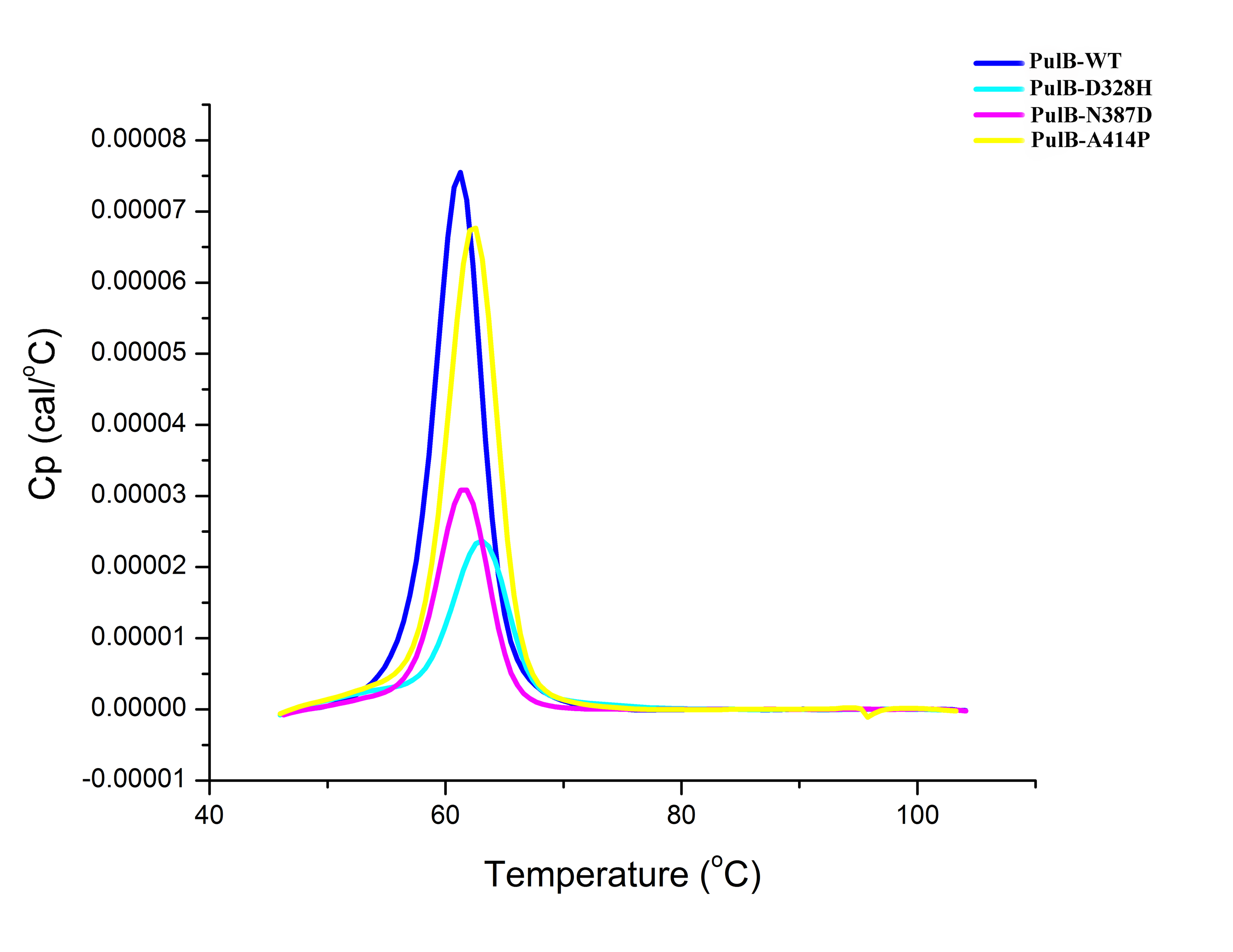

Supplement: S5 Fig — (TIF) [file pone.0165006.s005.tif]

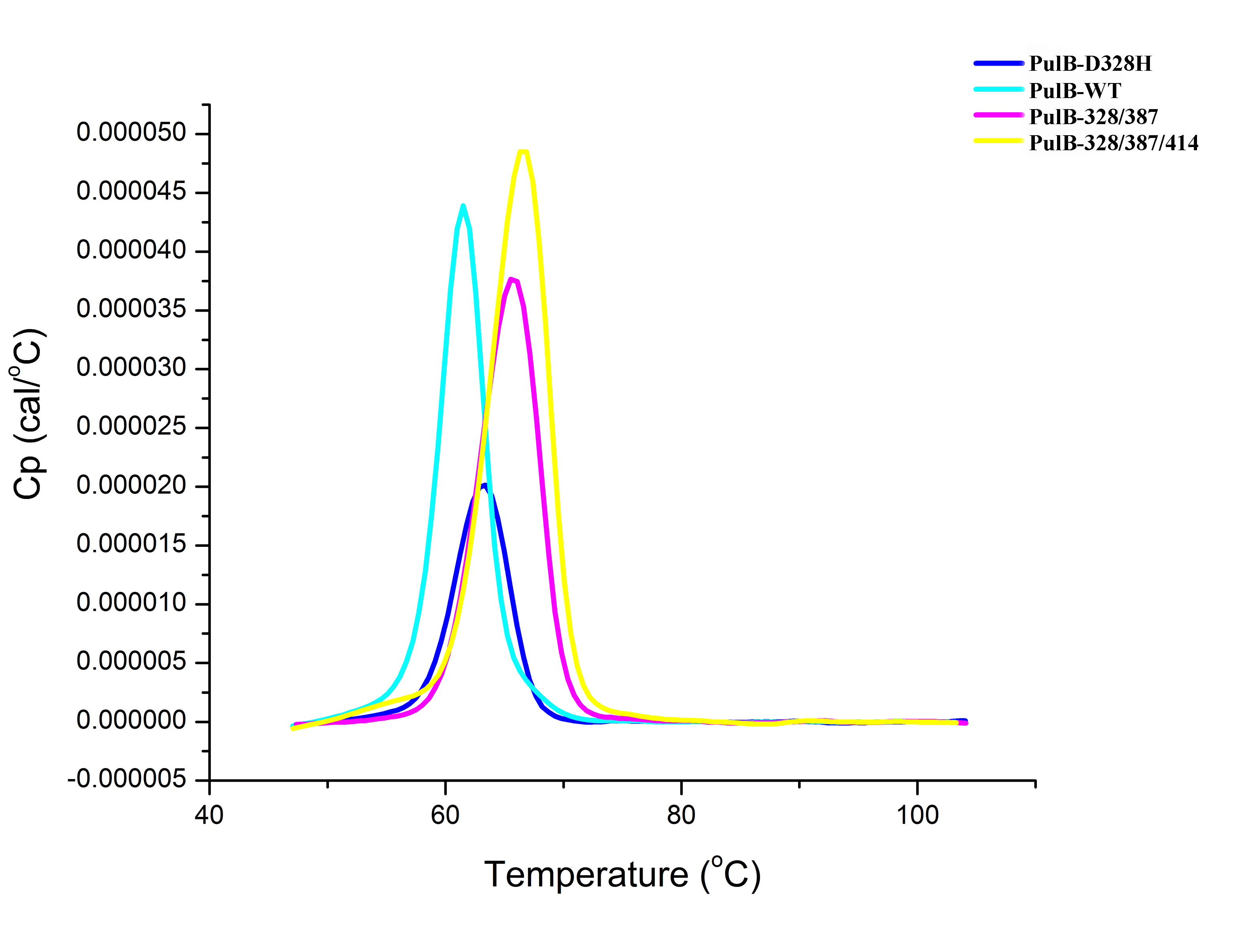

Supplement: S6 Fig — (TIF) [file pone.0165006.s006.tif]
