# Supplementary material for: Improving the Thermostability of Acidic Pullulanase from Bacillus naganoensis by Rational Design
Source: PLoS One. 2016 Oct 20;11(10):e0165006. doi: 10.1371/journal.pone.0165006 (PMC5072709; doi:10.1371/journal.pone.0165006)
Supplement: S1 Table — (DOCX) [file pone.0165006.s007.docx]

**S1 Table. Sequences of the primers used in this study.**

| Forward Primer | Sequence |
| --- | --- |
| pul-WT-F | 5’-CCTGCTGTAAGTAACGCTTATTTAGATGC-3’ |
| pul-WT-R | 5’-TTTACCATCAGATGGGCTTACTTCTTGATG-3’ |
| pul-287F | 5’-CGTATGCAACAAAGATTGCACCAAACG-3’ |
| pul-287R | 5’-TGCAATCTTTGTTGCATACGGATCAAC-3’ |
| pul-328F | 5’-GTCATCTATGAAATGCACGTTCGTGAC-3’ |
| pul-328R | 5’-GTCACGAACGTGCATTTCATAGATGA-3’ |
| pul-387F | 5’-ATTTAATAGTGTCGATGAAAACGATCC-3’ |
| pul-387R | 5’-GGATCGTTTTCATCGACACTATTAAAT-3’ |
| pul-414F | 5’-TGCTACTAATCCTAACGGAACAACTCG-3’ |
| pul-414R | 5’-TGTTCCGTTAGGATTAGTAGCATATTG-3’ |
| pul-662F | 5’-CCTCACAAGGCGTACCTTTCATGCAGG-3’ |
| pul-662R | 5’-CCTGCATGAAAGGCATGCCTTGTGAGG-3’ |
| pul-687F | 5’-TGGTGATTCAGTGAACGAGTTTGATTG-3’ |
| pul-687R | 5’-ATCAAACTCGTTCACTGAATCACCAGC-3’ |
| pET22b-F | 5’-TTACTGATGATGAACATGCCC-3’ |
| pET22b-R | 5’-GTTCATCATCAGTAACCCGTA-3’ |
