# Supplementary material for: Improving the Thermostability of Acidic Pullulanase from Bacillus naganoensis by Rational Design
Source: PLoS One. 2016 Oct 20;11(10):e0165006. doi: 10.1371/journal.pone.0165006 (PMC5072709; doi:10.1371/journal.pone.0165006)
Supplement: S1 Text — (PDF) [file pone.0165006.s008.pdf]

```
                                1                                                           60
                     Pul-WT    (1) ------------------------------------------------------------
Bacillus acidopullulyticus     (1) DSTSTKVIVHYHRFDSNYTNWDVWMWPYQPVNGNGAAYQFTGTNDDFGAVADTQVPGDNT
       Bacillus sp. CICIM 263  (1) -----------------------------------------------------MVQGIHD
   Thermotoga maritima MSB8    (1) ------------------------------------------------------------
      Thermotoga neapolitana   (1) ------------------------------------------------------------

                                   61                                                         120
                     Pul-WT    (1) ------------------------------------------------------------
Bacillus acidopullulyticus    (61) QVGLIVRKNDWSEKNTPNDLHIDLAKGHEVWIVQGDPTIYYN-----------LSDAQAA
       Bacillus sp. CICIM 263  (8) EVGFIVRTDSWEKDGGDRFINVQ-SGNDEVWVKAGDEHTYTSPPDGEYKDFPAFERVKVK
   Thermotoga maritima MSB8    (1) -------------------------MKTKLWLLLVLLLSALI-----------FSETTIV
      Thermotoga neapolitana   (1) -------------------------MKTRFWILLVLLFAVLI-----------FSETTII

                                   121                                                        180
                     Pul-WT    (1) --PAVSNAYLDASNQVLVKLSQPFTLGEGSSGFTVHDDTANKDIPVTSVSDANQVTAVLA
Bacillus acidopullulyticus   (110) AIPSVSNAYLDDEKTVLAKLSMPMTLADAASGFTVIDKTTGEKIPVTSAVSANPVTAVLV
       Bacillus sp. CICIM 263 (67) LHYFRYDGNYEGWNLWTWPEGKDGKRVD----FTAEDDFGKVAEFELENQEGMKRAGFIV
   Thermotoga maritima MSB8   (25) VHYHRYDGKYDGWNLWIWPVEPVSQEGK-AYQFTGEDDFGKVAVVKLPMDLTKVGIIVRL
      Thermotoga neapolitana  (25) VHYHRYDGKYDGWNLWIWPVEPVSQEGK-AYQFTDEDDFGKVAVVKLPLDLTTVGIIVRL

                                   181                                                        240
                     Pul-WT   (59) GTFQHIFGGS--------DWAPDNHNTLLKKVNSNLYQFSGNLPEGNYQYKVALNDSWNN
Bacillus acidopullulyticus   (170) GDLQQALGAAN-------NWSPDDDHTLLKKINPNLYQLSGTLPAGTYQYKIALDHSWN-
       Bacillus sp. CICIM 263(123) RKSMEGNDWAGREFGDRFITKFDENGNAEIWIVQGTESIYYNPDYIEKDPKIVNASMDT-
   Thermotoga maritima MSB8   (84) NEWQAKDVAKD-------RFIEIKDGKAEVWILQGVEEIFYEKPDTSPRIFFAQARSNK-
      Thermotoga neapolitana  (84) NEWQAKDVAKD-------RFIEIKDGKAEVWILQGVEEIFYEKPDTSPRIFFAQARSNK-

                                   241                                                        300
                     Pul-WT  (111) PSYPSDNINLTVPAGGAHVTFSYIPSTHAVYDTINNPNADLQVDSSGVKTDLVAVTLG--
Bacillus acidopullulyticus   (222) TSYPGNNVSLTVPQGGEKVTFTYIPSTNQVFDSVNHPNQAFPTSSAGVQTNLVQLTLA--
       Bacillus sp. CICIM 263(182) ----FNQITLETNFPFQWRDWKSIINIDNADIKEVVPYDGNEESNVTNKVKVVTEDKLDF
   Thermotoga maritima MSB8  (136) ----VIEAFLTNPVDTKKKELFKVTVDGKEIPVSRVEKADPTDIDVTNYVRIVLSESL--
      Thermotoga neapolitana (136) ----VIEAYLTNPADTKKKGLFKVTVDGKEIPISRVEKADPTDVDVTNYVRVVLSEPL--

                                   301                                                        360
                     Pul-WT  (169) ENPDVSHTLSIQTEDYQAGQVIPRKVLDSSQYYYSGDDLGNTYTKNATTFKVWAPTSTQV
Bacillus acidopullulyticus   (280) SAPDVTHNLDVAADGYKAHNILPRNVLNLPRYDYSGNDLGNVYSKDATSFRVWAPTASNV
       Bacillus sp. CICIM 263(238) RQSYKISAVSFGEAEVKIGEIVRSKEFDDAFYYDG--KLGNQYTKNKTSFRLWAPTASEA
   Thermotoga maritima MSB8  (190) KEEDLRKDVELIIEGYKPARVIMMEILDDYYYDG---ELGAVYSPEKTIFRVWSPVSKWV
      Thermotoga neapolitana (190) KEEDLRKDVELIVEGYKPARVIMMEILDDYYYDG---ELGAIYSPERTIFRVWSPVAKWA

                                   361                                                     420 287
                     Pul-WT  (229) NVLLYNS-ATGAVTKTVPMTASGHGVWEATVNQDLENWYYMYEVTGQGSTRTAVDPYATA
Bacillus acidopullulyticus   (340) QLLLYNS-EKGSITKQLEMQKSDNGTWKLQVSGNLENWYYLYQVTVNGTTQTAVDPYARA
       Bacillus sp. CICIM 263(296) SIVLYDS-WDDQTAEELPLKRGEKGTWTTVLKGDQNGLIYNYKVKIGGEWTEAADPYVRA
   Thermotoga maritima MSB8  (247) KVLLFKNGEDTEPYQVVNMEYKGNGVWEAVVEGDLDGVFYLYQLENYGKIRTTVDPYSKA
      Thermotoga neapolitana (247) KVLLFRSGDDTEPFKEVDMDYKGNGVWEAVVEGNLDGVFYLYQLESYGKVRTTVDPYSKA

                                   421                                     328              480
```

```
                    Pul-WT  (288) IAPNGTRGMIVDLAKTDPAGWESDKHITPKNIEDEVIYEMDVRDFSIDSNSGMKNKGKYL
 Bacillus acidopullulyticus (399) ISVNATRGMIVDLKATDPAGWQGDHEQTPANPVDEVIYEAHVRDFSIDANSGMKNKGKYL
     Bacillus sp. CICIM 263 (355) VTVNGDRGVVMDLDSTNPKQWNKQK-PELKNPEDSIIYEVHVRDLSIHEDSGIMHKGKFL
  Thermotoga maritima MSB8  (307) VYANSKKSAVVNLARTNPEGWENDRGPKIEGYEDAIIYEIHIADITGLENSGVKNKGLYL
     Thermotoga neapolitana (307) VYANSKKSAVVDLSKTNPEGWESDKRPQIGGYEDAIIYEIHIADITGFENSGVKNRGLYL
                                  481                                     387           540
                    Pul-WT  (348) ALTEKGTKGPDNVKTGVDSLKQLGITHVQLQPVFAFNS---VNENDPTQYNWGYDPRNYN
 Bacillus acidopullulyticus (459) AFTEHGTKGPDHVKTGIDSLKELGITTVQLQPVEEFNS---IDETQPDTYNWGYDPRNYN
     Bacillus sp. CICIM 263 (414) GAAEKSTINSKGEKTGLNHIKDLGVTHVQFLPIYDYRT-VDETKLDEPQYNWGYDPKNYN
  Thermotoga maritima MSB8  (367) GLTEENTKGPGGVTTGLSHLVELGVTHVHILPFFDFYTGDELDKDFEKYYNWGYDPYLFM
     Thermotoga neapolitana (367) GLTEEGTKGPGGVTTGLPHLVELGVTHVHILPFFDFYTGDELDKDFEKYYNWGYDPYLFM
                                  541      414                                         600
                    Pul-WT  (405) VPEGQYATNANG-TTRIKEFKEMVLSLHQDHIGVNMDVVYNHTFAT-QISDFDKIVPEYY
 Bacillus acidopullulyticus (516) VPEGAYATTPEG-TARITELKQLIQSLHQQRIGVNMDVVYNHTFDV-MVSDFDKIVPQYY
     Bacillus sp. CICIM 263 (473) VPEGSYSTDPYDPAARITELKTMIQAYHDEQLRVVMDVVYNHVFAV-NESSFHKLVPGYY
  Thermotoga maritima MSB8  (427) VPEGRYSTDPKNPHTRIREVKEMVKALHKHGIGVIMDMVFPHTYGIGELSAFDQTVPYYF
     Thermotoga neapolitana (427) VPEGRYSTDPRNPYARIREVKEMVKALHRHDIGVIMDMVFPHTYGIGELSAFDQTVPYYF
                                  601                                                  660
                    Pul-WT  (463) YRTDDAGNYTNGSGTGNEIAAERPMVQKFIIDSLKFWVNEYHVDGFRFDLMALLGKDTMS
 Bacillus acidopullulyticus (574) YRTDSNGNYTNGSGCGNEFATEHPMAQKFVLDSVNYWVNEYHVDGFRFDLMALLGKDTMA
     Bacillus sp. CICIM 263 (532) FRYNEDGTLANGTGVGNDTASERKMVQKFIVDSVAYWAEEYNLDGFRFDLMGIHDTETMN
  Thermotoga maritima MSB8  (487) YRIDKTGAYLNESGCGNVIASERPMMRKFIVDTVTYWVKEYHIDGFRFDQMGLIDKKTML
     Thermotoga neapolitana (487) YRIDKTGAYLNESGCGNVIASERPMMRKFIVDTVTYWVKEYHIDGFRFGQMGLIDKKTML
                                  661                                                  720
                    Pul-WT  (523) KAATQLHAIDPGIALYGEPWTGGTSALPADQLLTKGAQKGMGVAVFNDNLRNGLDGSVFD
 Bacillus acidopullulyticus (634) KISNELHAINPGIVLYGEPWTGGTSGLSSDQLVTKGQQKGLGIGVFNDNIRNGLDGNVFD
     Bacillus sp. CICIM 263 (592) KVRKELDTIDPTIIIIGEGWDLNTPLAAEKKANQKNAEDMPGIGHFNDGIRDGLKGSVFD
  Thermotoga maritima MSB8  (547) EVERALHKIDPTIILYGEPWGG----WGAPIRFGKSDVAGTHVAAFNDEFRDAIRGSVFN
     Thermotoga neapolitana (547) EVEKALHKIDPTIILYGEPWGG----WGAPIRFGKNDVAGTHVAAFNDEFRDAIRGSVFN
                                  721                                                  780
                    Pul-WT  (583) SSAQGFATGATGLTDAIKNGVEGSIN-------DFTASPGETINYVTSHDNYTLWDKIAQ
 Bacillus acidopullulyticus (694) KTAQGFATGDPNQVDVIKNGVIGSIQ-------DFTSAPSETINYVTSHDNMTLWDKILA
     Bacillus sp. CICIM 263 (652) ELDKGFVNGKQGMESFVQQGIAAGLDYP--DEMATYKDPEQAVTYAEAHDNHTLWDKLEL
  Thermotoga maritima MSB8  (603) PSVKGFVMGGYGKETKIKRGVVGSINYDGKLIKSFALDPEETINYAACHDNHTLWDKNYL
     Thermotoga neapolitana (603) PSVKGFAMGGYGKETKIKRGVVGSINYDGKLIKSFASDPEETINYVACHDNHTLWDKNYL
                                  781                            662                 687 840
                    Pul-WT  (636) SNPND-----SEADRIKMDELAQAIVMTSQGIPFMQGGEEMLRTKGGNDNSYNAGDVVNE
 Bacillus acidopullulyticus (747) SNPSD-----TEADRIKMDELAHAVVFTSQGVPFMQGGEEMLRTKGGNDNSYNAGDSVNQ
     Bacillus sp. CICIM 263 (710) TNPDA-----EEEFKKKMHKLASSIILTSQGVSFVHAGQEFMRTKYGDHNSYKSPDSINQ
  Thermotoga maritima MSB8  (663) AAKADKKKEWTEEELKNAQKLAGAILLTSQGVPFLHGGQDFCRTKNFNDNSYNAPISING
     Thermotoga neapolitana (663) AAKADKRKKWTEEELKNAQKLSGAIILTSQGVAFLHGGQDFCRTKNFNDNSYNAPISING
                                  841                                                  900
                    Pul-WT  (691) FDWSRKAQYPDVFNYYSGLIHLRLDHPAFRMTTANEINSHLQFLNSPENTVAYELSDHAN
 Bacillus acidopullulyticus (802) FDWSRKAQFKDVFDYFSSMIHLRNQHPAFRMTTADQIKQNLTFLESPTNTVAFELKNYAN
```

Bacillus sp. CICIM 263	(765)	LDWDRRTEFSGEVDYFKGLIKLRKHYKSFRMTTAEDIQSKLHFIDAPDNTVAYRLDAKGL
Thermotoga maritima MSB8	(723)	FDYERKLQFIDVFNYHKGLIKLRKEHPAFRLKNAEEIKKHLEFLPGGRRIVAFMLKDHAG
Thermotoga neapolitana	(723)	FGYERKRQFIDVFNYYKGLIKLRKEHPAFRLKTAEEIKKHLEFLPGGRRIVAFILKDHAG

		901 ... 960
Pul-WT	(751)	KDTWGNIVVIYNPNKTAETINLP-SGKWEINATSGKVGESTLGQAEG-SVQVPGISMMIL
Bacillus acidopullulyticus	(862)	HDTWKNIIVMYNPNKTSQTLNLP-SGDWTIVGLGDQIGEKSLGHVMG-NVQVPAISTLIL
Bacillus sp. CICIM 263	(825)	KDRAKEIVVIHNANTEPAKISLPGKGPWHLLADGKQAGIRTLKIYQSKTIEVPAQTSFIL
Thermotoga maritima MSB8	(783)	GDPWKDIVVIYNGNLEKTTYKLP-EGKWNVVVNSQKAGTEVIETVEG-TIELDPLSAYVL
Thermotoga neapolitana	(783)	GDPWKDIVVIYNGNPEKSTYKLP-EGKWNLVVNGQKAGTEVIEVVEG-TIELEPLSAYVL

		961
Pul-WT	(809)	HQEVSPSDGK
Bacillus acidopullulyticus	(920)	KQ--------
Bacillus sp. CICIM 263	(885)	KR--------
Thermotoga maritima MSB8	(841)	YRE-------
Thermotoga neapolitana	(841)	YRE-------

S1 Text.

Sequence alignment between Pul-WT and *Bacillus acidopullulyticus, Bacillus sp.* CICIM 263, *Thermotoga maritima* MSB8, *Thermotoga neapolitana*
